# The Specific Impact of Apolipoprotein E Epsilon 2 on Cognition and Brain Function in Cognitively Normal Elders and Mild Cognitive Impairment Patients

**DOI:** 10.3389/fnagi.2019.00374

**Published:** 2020-01-28

**Authors:** Liang Gong, Ronghua Xu, Duan Liu, Lin Lan, Bei Zhang, Chuantao Zhang

**Affiliations:** ^1^Department of Neurology, Chengdu Second People’s Hospital, Chengdu, China; ^2^Hospital of Chengdu University of Traditional Chinese Medicine, Chengdu, China

**Keywords:** apolipoprotein E, Alzheimer’s disease, functional connectivity density, imaging genetics, hippocampus

## Abstract

Variants in the apolipoprotein E (*APOE*) gene play an important role in the development of Alzheimer’s disease (AD). Specifically, the *APOE* ε4 allele is an established genetic risk factor for AD, while the *APOE* ε2 allele is a protective factor against AD. However, the mechanism underlying this impact of *APOE* genotype on the pathogenesis of AD remain unclear. This study sought to investigate the influence of *APOE* genotype on cognition and neuroimaging features in cognitively normal (CN) elderly individuals and patients with mild cognitive impairment (MCI). A total of 177 participants were selected from the Alzheimer’s Disease Neuroimaging Initiative (ADNI) database, including 101 MCI patients and 76 CN individuals. A 2 × 3 (consisting of two groups and three *APOE* genotypes) analysis of covariance was carried out to measure the influences of diagnosis and *APOE* genotype on cognition and brain features, assessed based on global functional connectivity density (gFCD) and hippocampal volume. In addition, a mediation analysis was carried out to investigate the indirect influence of neuroimaging features on the relationship between *APOE* genotype and cognitive performance in the MCI group. This analysis revealed that *APOE* genotype had an influence on brain function in the bilateral precentral gyrus, right thalamus, and posterior cingulate cortex (PCC). In addition, an interactive influence between diagnosis and *APOE* genotype was found on general cognition, immediate memory, executive function, hippocampal volume, and gFCD in the right dorsolateral prefrontal cortex and medial prefrontal cortex (MPFC). Finally, this mediation analysis revealed that hippocampal volume and gFCD in the thalamus may mediate the relationship between *APOE* genotype and cognitive performance in the MCI group. Taken together, our findings provide novel insights into the neural mechanisms underlying the genetically guided pathogenic mechanisms of AD.

## Introduction

Apolipoprotein E (*APOE*) is a major genetic risk factor contributing to the development of late-onset Alzheimer’s disease (AD). Individuals with the ε4 allele have increased risks, while those with the ε2 allele show decreased risk of developing AD (Seripa et al., 2009; Fei and Jianhua, 2013; Liu et al., 2013). However, the neuropathological mechanism underlying the opposite outcomes on cognitive function is still unclear. In the last few decades, many studies investigated the impact of *APOE* genotype variations on brain structure and function in individuals with AD or mild cognitive impairment (MCI) and healthy elders using neuroimaging and genetic analysis (Spampinato et al., 2011; Troyer et al., 2012; Wang et al., 2015). Most of the studies focused on the neural basis of the *APOE* ε4 allele and investigated the group differences in brain structure and function between ε4 and non-ε4 carriers. Because of the relative rarity of the ε2 allele, studies examining the influences of the *APOE* ε2 allele on the neuropathology of AD are still lacking, and the mechanisms underlying the impact of the ε2 allele on AD development remain unclear (Suri et al., 2013).

With recent advances in neuroimaging, genetic imaging approaches have been widely used to explore the genetic impact on the brain, of which structural MRI and functional MRI (fMRI) are the most widely used technologies. A large body of work has focused on the relationship between *APOE* genotype and AD pathology. Many prior studies have examined the structural and functional differences in the brain, particularly in the hippocampus, resulting from the presence of ε4 (Schuff et al., 2009; Wang et al., 2015; Sinclair et al., 2017). More recently, researchers have paid more attention to the impact of the *APOE* ε2 allele on the brain. Using both task-based and resting-state fMRI (rs-fMRI), Trachtenberg et al. (2012a; 2012b). found that hippocampus activation and functional connectivity were similarly changed in ε4 and ε2 carriers when compared with those in ε3 carriers in cognitively normal (CN) elders. Shu et al. (2014) also demonstrated that *APOE* ε2 and ε4 carriers both showed decreased functional connectivity compared with carriers of homozygous ε3 in CN elders. However, Chen et al. (2016) found opposite outcomes of *APOE* ε2 and ε4 on the intrinsic entorhinal cortex functional connectivity in the middle temporal gyrus. Altered functional connectivity was correlated with episodic memory in the preclinical stage of AD (amnestic MCI) (Chen et al., 2016). In addition, the influences of *APOE* genotypes on hippocampal volumes have been frequently examined. Hostage et al. (2013) found dose-dependent influences of ε4 alleles on hippocampal volumes in AD and MCI patients, as well as those of ε2 alleles in CN elders. Therefore, the hippocampus is the most widely studied and affected part of the brain in early stages of AD (Schuff et al., 2009). However, whether and how altered hippocampus volume and brain function is implicated in the opposite role of *APOE* genotypes in cognitive performance in the development of AD remains unclear.

In the present study, we used global functional connectivity density (gFCD) mapping, a new data-driven, voxel-wise method, to measure the function of the whole brain based on rs-fMRI data. We investigated the impact of the *APOE* genotype (ε2+, ε3/ε3, and ε4+) and its interaction with disease status on cognitive function, hippocampal volume, and gFCD in CN elderly and MCI patients. Importantly, mediation analysis was used to explore potential intermediate phenotypes in the relationship between the *APOE* genotype and cognitive performance in the CN and MCI population. We hypothesized that *APOE* ε2 will have a particular impact on cognitive performance, hippocampal volume, and brain function in CN elders and MCI participants compared to the *APOE* ε4 allele. In addition, we hypothesized that the gene–brain–cognition model may potentially be able to elucidate the mechanisms underlying *APOE*-associated cognitive impairment in the MCI group.

## Materials and Methods

### The Alzheimer’s Disease Neuroimaging Initiative

Data used in this study were obtained from the Alzheimer’s Disease Neuroimaging Initiative (ADNI) database^1^. The database was launched in 2003 as part of a public–private partnership led by principal investigator Michael W. Weiner, MD, United States. The primary goal of ADNI is to test whether serial MRI, PET, other biological markers, and findings of clinical and neuropsychological examinations can be combined to assess the progression of MCI and early AD. Updated information can be obtained at www.adni-info.org. Ethical approval was obtained by the investigators of the ADNI^2^. The institutional review boards of the respective institutions at all participating sites approved the study. All ADNI participants provided written informed consent before the start of the study.

### Participants

The inclusion and exclusion criteria for all ADNI participants are available at http://www.adni-info.org. Participants were selected in the present study according to the following inclusion criteria: Caucasian ethnicity, availability of rs-fMRI and 3D T1-weighted MRI images, and underwent genotyping of the *APOE* gene. A battery of neuropsychological tests were used in this study, including the Mini-Mental State Examination (MMSE), AD assessment scale – 13-item cognitive subscale (ADAS13) for general cognition. The Rey Auditory Verbal Learning Test (RAVLT) is a powerful tool for assessing memory and learning (Schmidt, 1996). In this study, we used the RAVLT-immediate (sum of scores of trials 1–5) to assess participants’ immediate memory, the RAVLT learning (score of trial 5 minus trial 1) to assess learning, and the RAVLT-forgetting (score of trial 5 minus the score of delayed recall) and Logical Memory test (delayed recall score) to assess delay memory. The scores of the Trails *B* test were used to assess participants’ executive function. The naming of the neuropsychological test used in our study will be available from the merged table provided by the ADNI, and the derived summary scores obtained from the RAVLT measurements can be found in the ADNI data dictionary^3^. The diagnosis of MCI was based on the guidelines described in the ADNI protocol, including the following: (1) complaints with regard to memory-related problem or observation of such problems by a partner; (2) a specified education-adjusted cutoff score on the Logical Memory test; an MMSE score between 24 and 30; a Clinical Dementia Rating score of 0.5; a Memory Box score of at least 0.5; and (3) relatively well-preserved daily life activities. Detailed inclusion and exclusion criteria are available at http://adni.loni.usc.edu/wp-content/uploads/2010/09/ADNI_GeneralProceduresManual.pdf. Accordingly, 185 participants were included in the first step; however, 8 participants were excluded because of poor signals in the rs-fMRI images (*N* = 3) or excessive head motion (translations >3 mm or rotation > 2°) (*N* = 5). Finally, the remaining 177 participants, including 76 CN elders and 101 patients with MCI, were included in the analysis.

### Genotyping

All selected participants underwent genotyping to assess their *APOE* allele status. *APOE* genotyping was performed using DNA extracted from peripheral blood cells which were collected in plastic tubes with ethylenediaminetetraacetic acid (10 ml). The University of Pennsylvania AD Biomarker Fluid Bank Laboratory will receive and store the biomarker samples, which will be processed at the University of Pennsylvania. More detailed information is available in the ADNI-1 Procedures manual^4^. In addition, five participants with the ε2/ε4 genotype were excluded from the analysis because of the opposing outcomes of the ε2 and ε4 alleles (Shaw et al., 2007). *APOE* ε2 is a rare genotype; therefore, our study had only one CN participant who was homozygous for APOE ε2. Therefore, the ε2 homozygotes and heterozygotes were pooled into a single ε2 + carrier category. Furthermore, the study had only six patients with MCI who were homozygous for APOE ε4, and five CN participants who were homozygous for APOE ε4. Therefore, we also pooled the ε4 homozygotes and heterozygotes into a single ε4 + carrier category. In total, 31 *APOE* ε2 + (ε2ε2/ε2ε3), 85 ε3ε3, and 60 ε4 + (ε3ε4/ε4ε4) participants were included in the final analysis.

### MRI Data Acquisition

All rs-fMRIs were performed using a single-shot T2 star-weighted echo-planar imaging pulse sequence with a Philips 3T MRI scanner with an eight-channel head coil. The rs-fMRI parameters were as follows: repetition time, 3,000 ms; echo time, 30 ms; flip angle, 80°; acquisition matrix, 64 × 64; field of view, 240 × 240 mm; thickness, 3.3 mm; gap, 0 mm; and number of slices, 48. A total of 140 image volumes were acquired in the rs-fMRI scans. In addition, 3D T1-weighted MRI images were obtained using a magnetization-prepared gradient echo sequence with a spatial resolution of 1 mm × 1 mm × 1.2 mm. The detailed MRI protocols can be found at http://adni.loni.usc.edu/methods/documents/mri-protocols/.

### Structural Image Analysis

Cortical reconstruction and volumetric segmentation were performed using the FreeSurfer version 5.1 image analysis suite^5^. The technical details for using the FreeSurfer with the ADNI data have been described by many studies previously (Holland et al., 2009; Chiang et al., 2010; Jack et al., 2010). Briefly, the image processing protocol included motion correction and registration, non-uniform intensity normalization, Talairach transform computation, intensity normalization 1, skull strip, segmentation of the subcortical white matter and the deep gray matter volumetric structures, tessellation of the gray matter/white matter boundary, automated topology correction, and surface deformation with intensity gradients to optimally place the gray/white and gray/cerebrospinal fluid borders at the location where the greatest shift in intensity defines the transition to other tissue class. The image data were processed based on the 2010 Desikan–Killiany atlas (Fischl et al., 2004). We selected the hippocampal and entorhinal cortical volumes for structural analysis, as these two regions are usually affected in the early stages of AD and MCI. The fusiform was selected as a reference region in the structural analysis. The bilateral hippocampal, entorhinal, and fusiform volumes were summed in the analyses to increase the statistical power of the analysis. Because an interactive outcome (diagnosis × *APOE*) was found in the hippocampal volume (see section “Results”), an additional analysis was carried out using the volumes of the left and right hippocampus separately (see the Supplementary Material for detailed information).

### Rs-fMRI Data Preprocessing

Functional data were preprocessed using the SPM12 toolkit^6^ and MATLAB version 7.10 (The MathWorks, Inc., Natick, MA, United States). The preprocessing steps were conducted using the BRAinNetome Toolkit software^7^. Structural images were segmented (VBM toolbox in SPM) and coregistered with the resting functional images. The fMRI images were preprocessed as follows: The first 10 volumes in the scanning session were discarded to attain equilibration. The remaining 130 volumes were corrected for slice timing, realigned, and subsequently normalized spatially using diffeomorphic high-dimensional registration as implemented in the DARTEL toolbox using default settings, and the volumes were resampled to 3 mm × 3 mm × 3 mm cubic voxels. The blood oxygenation level dependent signal was low-pass filtered (0.01–0.1 Hz) and detrended. We also calculated the frame-wise displacement (FD), which evaluates the mismatch of volume-to-volume superimposed head position (Power et al., 2013). The mean FD was also used as a covariate in the image analyses. The FD did not differ significantly among the different groups (*p* > 0.05). Subsequently, the six motion parameters and the white matter and cerebrospinal fluid signals were excluded from the data analysis by linear regression.

### gFCD Calculation

We calculated the gFCD of each voxel using an in-house script according to the method described by Tomasi and Volkow (2010). The gFCD at a given voxel (*x*_0_) was computed as the global number of functional connections using Pearson’s linear correlation between x0 and all the other voxels; two voxels with a correlation coefficient of >0.6 were considered functionally connected. In addition, the gFCD calculation was restricted to the gray matter regions with a signal/noise ratio of >50% to minimize unwanted effects from susceptibility-related signal-loss artifacts (Tomasi and Volkow, 2010). To increase the normality of the distribution, grand mean scaling of the gFCD values of each voxel was divided by the mean value of the qualified voxels of the whole brain. Finally, the normalized gFCD maps were spatially smoothed with 6 mm × 6 mm × 6 mm Gaussian kernels.

### Statistical Analyses

The 2 × 3 (two groups, three *APOE* genotypes) analysis of covariance (ANCOVA) and the Kruskal–Wallis tests were used to compare the demographic data, neuropsychological performances and structural brain maps among the different groups using the Statistical Package for the Social Sciences software version 24.0 (SPSS, Inc., Chicago, IL, United States). The effects of age, sex, and years of education were regressed out in the analyses of cognitive function, and the effect of intracranial volume was regressed out in the structural comparison of the brain. The statistical threshold was set at *p* < 0.05.

The voxel-wise comparisons of the global FCD maps were conducted using a 2 × 3 (diagnosis × *APOE* genotype) ANCOVA from the SPM12 toolkit with age, sex, years of education, and mean FD as the nuisance covariates. The voxel-level significant threshold was set at *p* < 0.005, corrected for multiple comparisons at the cluster level with the latest version of the 3dClustSim program in AFNI_16.3.00 software [gray matter mask correction (67,541 voxels), voxel-level *p* < 0.005, cluster level α < 0.001, κ > 50, cluster size >1,647 mm^3^]^8^. Subsequently, the average gFCD strength in each cluster was extracted from each subject using the masks generated from the ANCOVA analysis for quantitative illustration and additional mediation analyses.

Furthermore, the mediation analysis was used to examine whether the generated gFCD maps and brain volumes could mediate the influences of the *APOE* genotypes on cognitive performances in the MCI group. First, multivariate linear regression analyses were performed to explore the influence of the *APOE* genotype on cognitive performances in the MCI and CN groups, separately, after adjusting for the effects of age, sex, and years of education. Subsequently, the *APOE* genotype was set as an independent variable (*X*), and the *APOE* genotype associated with cognition performances (the MMSE and ADAS scores) was set as the dependent variables (*Y*). Because a significant association between *X* and *Y* is not a prerequisite to testing a mediation hypothesis (Hayes and Rockwood, 2017), other indices of cognitive performance, including the RAVLT, Trail B, and Logical Memory scores, were also set as dependent variables (*Y*). Second, the extracted gFCD values in the brain regions exhibiting the primary outcome of the *APOE* genotypes and the hippocampus volume were set as the mediators (*M*). Third, we used the simple mediation model from the PROCESS macro in SPSS (Model 4, version 2.16.3) (Hayes, 2013; Gong et al., 2017b) and adjusted the effects of sex, age, and years of education as covariates. This model was based on 10,000 bootstrap samples for assessing the bias-corrected bootstrap confidential interval (CI). The indirect influence was determined as significant at 95% CI, with a null hypothesis without indirect influence. Linear regression analyses showed no influence of the *APOE* genotypes on any cognitive performances in the CN group; therefore, the mediation analysis was only conducted in the MCI group.

## Results

### Demographic Information and Neuropsychological Characteristics

**FIGURE 1:**
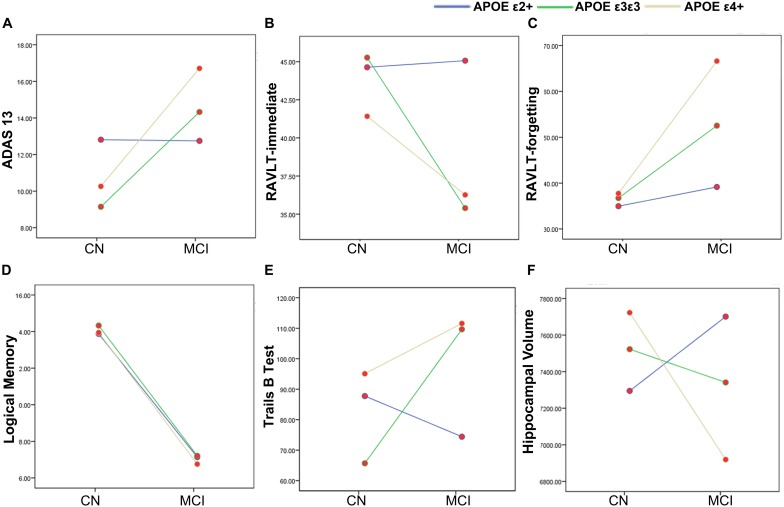
The main and interactive influences of diagnosis and apolipoprotein E (*APOE*) genotype on cognitive function and hippocampal volume. **(A,B,E,F)** Interactive influence of diagnosis and *APOE* genotype on the hippocampal volume and scores of ADAS13, RAVLT-immediate, and Trails *B* test. Compared to the *APOE* ε3ε3 and ε4 + genotypes, the *APOE* ε2+ genotype show more stable cognitive function and hippocampus volume during the CN to MCI period. **(C,D)** Main influences of diagnosis on the scores of RAVLT-forgetting and Logical Memory tests. The MCI group show lower logical memory and more RAVLT-forgetting rate as compared to the CN group. CN, cognitively normal; MCI, mild cognitive impairment; ADAS13, Alzheimer’s disease (AD) assessment scale–13-item cognitive subscale; RAVLT, Rey Auditory Verbal Learning Test.

Demographic information and neuropsychological evaluations for each group are shown in Table 1. In total, 31 *APOE* ε2 +(ε2ε2/ε2ε3), 85 ε3ε3, and 60 ε4+ (ε3ε4/ε4ε4) participants were included in the final analysis. There were no significant differences in age, sex, and years of education between the CN and MCI groups with different *APOE* genotypes (all *p* > 0.05). No significant main and interactive influences of diagnosis and *APOE* genotypes on intracranial volume and mean FD were found between the groups. Compared with the CN participants, patients with MCI had poor cognitive performances in ADAS13 (Figure 1A, *F* = 13.59, *p* < 0.001), RAVLT-immediate (Figure 1B, *F* = 9.54, *p* = 0.002), RAVLT-forgetting (Figure 1C, *F* = 7.71, *p* = 0.006), and Logical Memory (Figure 1D, *F* = 200.39, *p* < 0.001). The *APOE* genotype influence was found in RAVLT-immediate (*F* = 3.69, *p* = 0.02). *Post hoc* analysis revealed that the RAVLT-immediate score in the *APOE* ε2+ group (44.48) was higher than that in the *APOE* ε3/ε3 (39.22) and ε4+ (39.00) groups (*p* = 0.02), while the RAVLT-immediate score was similar in the ε3/ε3 and ε4+ carriers. In addition, the interactive influences of diagnosis and the *APOE* genotypes on cognitive function, as assessed by ADAS13 (*F* = 3.35, *p* = 0.03), RAVLT-immediate (*F* = 3.35, *p* = 0.03), and Trails *B* test (*F* = 4.57, *p* = 0.01), and the hippocampal volume (*F* = 5.78, *p* = 0.007) were observed. More specifically, as shown in Figures 1A,B,E, the interactive influence was mainly observed in the *APOE* ε2+ genotype and *APOE* ε4+ genotype between the CN and MCI groups. Participants with the *APOE* ε2+ genotype showed more stabilized cognitive function, including general cognition (ADAS13 score), immediate memory (RAVLT-immediate score), and executive function (Trails *B* test) in the progression from CN to MCI. In addition, the hippocampal volume in MCI with the *APOE* ε2+ genotype was higher than CN with *APOE* ε2+ genotype, while the hippocampal volume in MCI with the *APOE* ε4+ genotype was lower than that in CN participants with the *APOE* ε4+ genotype (Figure 1F). Notably, no significant main or interactive outcome of disease and *APOE* genotypes was found in the volumes of the entorhinal cortex and fusiform gyrus (all *p* > 0.05).

**TABLE 1:** Demographic, neuropsychological, and brain structural information in each group.

	CN			MCI			Diagnosis influence	*APOE influence*	Diagnosis × *APOE* influence
	*APOE* ε2+	*APOE* ε3ε3	*APOE* ε4+	*APOE* ε2+	*APOE* ε3ε3	*APOE* ε4 +	*p*-value	*p*-value	*p*-value
	(*n* = 19)	(*n* = 39)	(*n* = 18)	(*n* = 13)	(*n* = 46)	(*n* = 42)			
Age	72.05 ± 5.22	75.07 ± 6.39	71.99 ± 4.50	72.86 ± 7.10	72.20 ± 7.73	70.16 ± 6.81	0.25	0.10	0.42
Sex (F/M)	10/9	19/21	8/10	5/8	24/22	18/24	0.62	0.73	0.40
Years of education	16.89 ± 2.35	15.71 ± 2.59	17.39 ± 1.94	14.92 ± 3.30	15.95 ± 2.55	16.03 ± 2.97	0.46	0.58	0.11
ADAS13	11.89 ± 6.56	9.21 ± 3.94	9.25 ± 3.36	13.33 ± 7.97	13.81 ± 6.25	15.68 ± 6.71	<0.001	0.21	0.03
MMSE	28.63 ± 1.77	28.79 ± 1.39	28.78 ± 1.30	28.92 ± 1.44	28.10 ± 1.53	27.69 ± 1.94	0.16	0.15	0.07
RAVLT-immediate	45.47 ± 11.21	43.76 ± 10.05	42.44 ± 8.93	42.91 ± 11.71	35.37 ± 9.84	37.52 ± 10.07	0.002	0.02	0.03
RAVLT-learning	5.26 ± 2.02	5.69 ± 2.50	5.88 ± 2.65	4.91 ± 1.68	4.95 ± 2.91	4.40 ± 2.64	0.08	0.61	0.30
RAVLT-forgetting (%)	35.31 ± 28.82	39.40 ± 23.33	36.53 ± 23.54	42.09 ± 28.06	52.71 ± 30.67	64.77 ± 28.88	0.001	0.05	0.13
Logical memory	13.79 ± 3.67	13.76 ± 2.84	13.94 ± 2.64	6.61 ± 2.81	6.91 ± 2.81	6.71 ± 3.06	<0.001	0.72	0.94
Trails *B* Test (Second)	84.52 ± 45.11	72.84 ± 22.35	90.11 ± 61.41	81.07 ± 33.10	109.93 ± 60.54	105.90 ± 54.59	0.05	0.08	0.01
ICV (10^3^ml)	1.49 ± 0.13	1.57 ± 0.18	1.51 ± 0.16	1.51 ± 0.17	1.53 ± 0.15	1.51 ± 0.18	0.69	0.05	0.61
Hippocampus (ml)	7.32 ± 1.08	7.44 ± 0.90	7.74 ± 0.72	7.64 ± 0.52	7.43 ± 0.86	7.29 ± 1.13	0.19	0.63	0.007
Entorhinal (ml)	3.64 ± 0.96	3.96 ± 0.67	3.81 ± 0.75	3.84 ± 0.48	3.71 ± 0.55	3.47 ± 0.85	0.29	0.17	0.20
Fusiform (ml)	17.76 ± 1.96	18.28 ± 2.02	18.07 ± 2.41	18.23 ± 2.46	17.45 ± 1.83	17.62 ± 2.31	0.11	0.91	0.43

*Kruskal–Wallis test was used for sex comparisons. CN, cognitively normal; MCI, mild cognitive impairment; ADAS-13, Alzheimer’s disease assessment scale–13 items cognitive subscale. MMSE, Mini-Mental State Examination; RAVLT, Rey Auditory Verbal Learning Test; ICV, intracranial volume.*

### Main Influence of the Diagnosis and the APOE Genotype on gFCD

**FIGURE 2:**
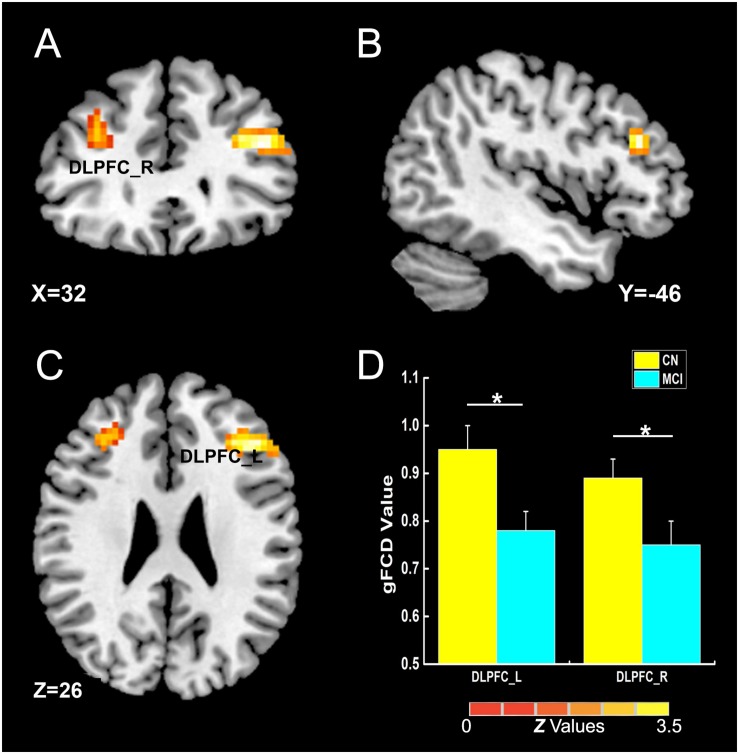
The main influence of diagnosis on gFCD (*p* < 0.005; α < 0.001; 3dClustSim corrected). **(A–C)** The brain regions show the differences in gFCD values between the two groups; **(D)** the histogram quantitatively illustrates that the values of gFCD in the bilateral DLPFC in the MCI group was lower than that in the CN group. The asterisks mean significant difference between two groups. gFCD, global functional connectivity density; CN, cognitively normal; MCI, mild cognitive impairment; DLPFC, dorsolateral prefrontal cortex.

**FIGURE 3:**
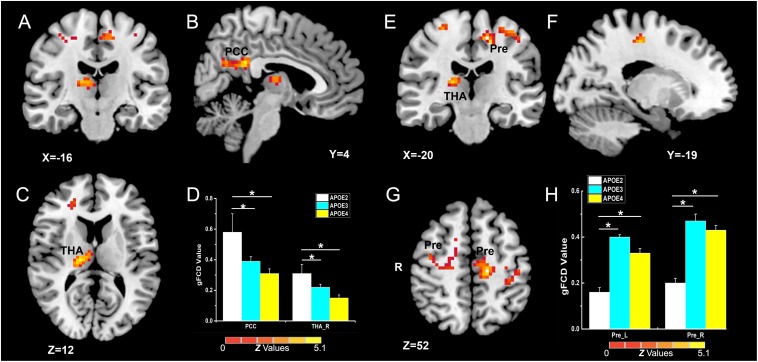
Primary outcome of the *APOE* genotypes on gFCD (*p* < 0.005; α < 0.001; 3dClustSim corrected). **(A–C,E–G)** Influence of *APOE* genotype on the brain functions of different brain regions. **(D,H)** The histograms quantitatively illustrate the gFCD differences among the three *APOE* genotypes. The asterisks mean significant difference between two *APOE* genotype groups. Compared to *APOE* ε3ε3 and ε4+ genotypes, the *APOE* ε2 + genotype show higher gFCD values in the PCC and right thalamus **(D)**, and lower gFCD values in the bilateral precentral gyrus **(H)**. No significant difference was found in brain functions between the two *APOE* ε3ε3 and ε4+ genotype groups. gFCD, global functional connectivity density; PCC, posterior cingulate cortex; THA, thalamus; Pre, precentral gyrus.

As illustrated in Figure 2 and Table 2, the main influence of diagnosis on the gFCD was found in the bilateral dorsolateral prefrontal cortex (DLPFC). Particularly, the gFCD of the bilateral DLPFC was lower in the MCI group than in the CN group. The main influence of the APOE genotype on the gFCD was found in the bilateral precentral gyrus, right thalamus, and posterior cingulate cortex (PCC) (Figures 3A–C,E–G), which is located in the posterior default mode network (DMN, PCC) and the sensorimotor network (SMN, thalamus, and precentral gyrus) (Biswal et al., 1995; Raichle, 2015). *Post hoc* analyses revealed that the ε2+ genotype was the primary factor affecting the brain function. Particularly, compared with ε3 and ε4 carriers, ε2 carriers showed a higher gFCD in the PCC and the right thalamus and a lower gFCD in the bilateral precentral gyrus (Figures 3D,H). No significant difference was found between the ε3 and ε4 carriers.

**TABLE 2:** Brain areas with significant diagnosis, *APOE*, and diagnosis × *APOE* influences on global functional connectivity density.

	Brain regions	Brodmann area	Cluster size (voxels)	MNI coordinates (*x*,*y*,*z*)	Peak *Z* score
Main influence of diagnosis	Left DLPFC	46	88	−42,33,24	3.50
	Right DLPFC	46	53	33,33,30	3.06
Main influence of *APOE*	Left precentral gyrus	4/6	160	-15,21,54	5.08
	Right precentral gyrus	6	81	27,−21,63	4.10
	PCC	6	81	6,−42,27	4.71
	Right thalamus	–	56	15,−18,12	4.73
Interactive influences of diagnosis × *APOE*	Right DLPFC	9/46	79	27,39,27	3.58
	Right MPFC	11	98	6,60,0	3.29

*DLPFC, dorsolateral prefrontal cortex; PCC, posterior cingulate cortex; MPFC, medial prefrontal cortex.*

### Interactive Influence Between the Diagnosis and the APOE Genotype on gFCD

**FIGURE 4:**
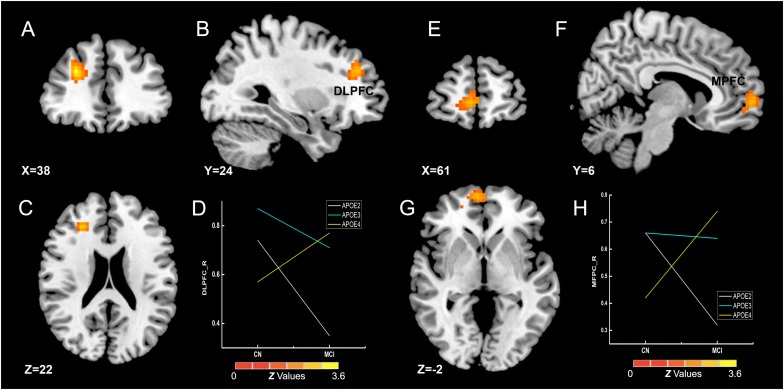
Interactive influence of diagnosis and *APOE* genotype on gFCD (*p* < 0.005; α < 0.001; 3dClustSim corrected). **(A–C,E–G)** Interactive influence of diagnosis and *APOE* genotype on the brain functions in the right DLPFC and MPFC. **(D,H)** The diagrams illustrate the interactive influence of diagnosis and *APOE* genotype in the DLPFC and MPFC. Compared to the CN group, the gFCD values in the DLPFC and MPFC was higher in patients with MCI who had the *APOE* ε2+ genotype and was lower in those who had the *APOE* ε4+ genotype; no significant alteration was noted in the MCI group with the *APOE* ε3ε3 genotype. gFCD, global functional connectivity density; CN, cognitively normal; MCI, mild cognitive impairment; DLPFC, dorsolateral prefrontal cortex; MPFC, medial prefrontal cortex.

The interactive influence between the diagnosis and the APOE genotype on gFCD was observed in the right DLPFC and medial prefrontal cortex (MPFC) (Figures 4A–C,E–G), which is located in the executive control network (ECN) (DLPFC) (Vincent et al., 2008) and anterior DMN (MPFC) (Raichle, 2015). As shown in Figures 4D,H, the interaction was observed in the alteration line between the APOE ε2 and ε4 carriers. Particularly, when compared with the CN group, the gFCDs in the right DLPFC and MPFC were decreased in MCI with APOE ε2 genotypes, but increased in MCI with ε4 carriers.

### Mediation Analyses

**FIGURE 5:**
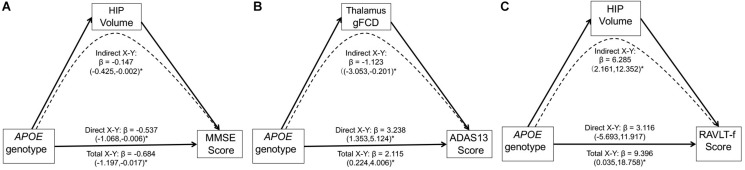
Mediation analyses show that the hippocampal volume and gFCD of the thalamus mediate the relationship between the *APOE* genotypes and cognitive performance in the MCI group. The asterisks mean the direct/indirect effect is significant. **(A)** The hippocampal volume negatively mediates the influence of *APOE* genotypes on the MMSE score in the MCI group. **(B)** gFCD values in the right thalamus negatively mediates the influence of *APOE* genotype on the ADAS score in the MCI group. **(C)** The hippocampal volume positively mediates the influence of *APOE* genotype on the RAVLT-forgetting score in the MCI group. gFCD, global functional connectivity density; MCI, mild cognitive impairment; HIP, hippocampus; MMSE, Mini-Mental State Examination; ADAS, Alzheimer’s disease assessment scale; RAVLT, Rey Auditory Verbal Learning Test.

Multivariate linear regression analyses revealed significant relationships between the APOE genotypes and MMSE scores (*F* = 4.69, *p* = 0.03) or ADAS 13 scores (*F* = 4.83, *p* = 0.01) in the MCI group. No other significant association between the APOE genotype and cognition function was found in either the MCI and CN group (all *p* > 0.05). We assessed the potential mediating role of the gFCD in APOE genotype-associated regions and hippocampal volumes in the relationship between the APOE genotype and cognitive performance in the MCI group. As shown in Figure 5, three significant mediators were found in the study. First, the hippocampal volume negatively mediated the APOE genotype influence on the MMSE score [Figure 5A, indirect influence, β = -0.147, 95% CI = (−0.425, −0.002)]. Second, the gFCD in the right thalamus also negatively mediated the APOE genotype influence on ADAS scores in MCI patients [Figure 5B, indirect influence, β = −1.123, 95% CI = (−3.053, −0.201)]. Third, the hippocampal volume could positively mediate the APOE genotype influence on the RAVLT-forgetting score in the MCI group [Figure 5C, indirect influence, β = 6.285, 95% CI = (2.161, 12.352)], although the direct influence of the APOE genotype on the RAVLT-forgetting score is not significant. No other significant mediator was found in the mediation analyses.

## Discussion

The present study aimed to explore the impact of *APOE* genotypes, especially the opposite ε2 and ε4 alleles, on cognitive function and brain in the CN and MCI groups. Four main findings were observed in our study. First, the *APOE* ε2 allele had a protective outcome on immediate memory, executive function, and hippocampal volume in the development from normal aging to MCI. Second, compared to *APOE* ε4 carriers, *APOE* ε2 carriers showed increased gFCD in the thalamus network, posterior DMN, and decreased gFCD in motor network. Third, the diagnosis state and *APOE* genotype synergistically contribute to brain functional alterations, especially in executive control network (ECN) and anterior DMN. Fourth, using the gene–brain–cognition model, we found that the hippocampal volume and thalamus function mediated the *APOE* genotype-associated cognitive performance in MCI patients. Taken together, these findings further extend our understanding of the influence of different *APOE* genotypes on cognition and brain and suggest that the gene–brain–cognition model may be used to determine the complex neural mechanisms underlying the pathogenesis and progression of AD.

The association between *APOE* ε4 and cognitive decline has been demonstrated in AD, MCI, and normal aging in many studies (Whitehair et al., 2010; Wisdom et al., 2011; Prada et al., 2014; Suarez et al., 2014), and the protective outcome of *APOE* ε2 on cognitive decline has been observed in AD patients (Corder et al., 1994; Suri et al., 2013). However, the positive outcome of the ε2 allele on cognitive function has not been replicated in healthy individuals. The present largest single-sample study has not found any association between the ε2 allele and special cognitive ability across all age ranges (Marioni et al., 2016). Sinclair et al. (2017) found that the ε2 allele showed positive outcomes on episode memory and executive function in early to mid-adult life (ages 23–67). In the present study, the *APOE* genotype was associated with general cognitive performance (MMSE and ADAS13 scores) in the MCI group, but was not associated with any cognitive domains in CN elders or MCI patients; this can be attributed to the small sample size in this study. Interestingly, the interactive influence of diagnosis and *APOE* genotype was found on the measure of immediate memory (RAVLT-immediate) and executive function (Trails *B* test) (Figures 1B,E). Both immediate memory and executive function are thought to be dependent on the brain function in the regions including the hippocampus and prefrontal cortex (Penney, 1989; Zelazo, 2015). These findings indicate the influence of *APOE* genotype on special cognition domains and brain regions during AD progression.

Changes in hippocampal volume serve as a sensitive and early biomarker of neurodegeneration in AD patients, and *APOE* ε4 is associated with atrophic hippocampal volume in AD (Liu et al., 2015) and MCI patients, but not in CN elders (Farlow et al., 2004; Morra et al., 2009). A previous multicohort study found a linear reduction in hippocampal volumes in AD and MCI patients with different *APOE* genotypes, i.e., ε4 carriers < ε3 carriers < ε2 carriers (Khan et al., 2017). Our study did not find differences in the hippocampus between the CN and MCI groups and the influence of *APOE* on hippocampal volume in the pooled group. However, the interactive influence of diagnosis and gene was found in the hippocampal volume (Figure 1F), and the result indicated that the ε2 and ε4 alleles had different influences at CN and MCI stages. The opposite outcomes of ε2 and ε4 on hippocampal volumes also point to the early pathological underpinnings of the *APOE* genotypes on cognitive function.

Consistent with previously reported abnormal brain functions in MCI patients, the MCI patients showed dysfunctional gFCD in the bilateral DLPFC (Wu et al., 2014), the core hub in the ECN (Vincent et al., 2008). These results support the idea that the abnormal functional connectivity in ECN may be an early biomarker of AD progression. The influence of *APOE* genotype on the intrinsic functional brain was found in the SMN (the bilateral precentral gyrus and thalamus) and posterior default mode network (PCC) (Biswal et al., 1995; Raichle, 2015; Figure 3). A previous study reported that the *APOE* ε2 carriers have robust white matter integrity (fractional anisotropy) in the right thalamus when compared to ε3/ε3 carriers in CN elderly individuals (Chiang et al., 2012) and that the *APOE* ε4 allele shows additive gray matter volume reduction in the precentral gyrus (Cacciaglia et al., 2018). Our findings showed the influence of *APOE* on brain structure, and the reverse influence of *APOE* ε2 and ε4 on SMN might indicate a counterbalance of brain function in SMN in the preclinical stage of AD. Regarding the impact of *APOE* on DMN, some studies showed increased DMN in young ε4 carriers and decreased DMN in older ε4 carriers (Filippini et al., 2009; Sheline et al., 2010), but other studies found similar DMN alterations in ε4 and ε2 carriers among CN elderly individuals and MCI patients (Trachtenberg et al., 2012a; Shu et al., 2014; Gong et al., 2017a). In our study, the gFCD in the posterior DMN was higher in ε2 carriers than in ε4 carriers. It is possible that the *APOE* genotype has different influences at different ages and disease development stages. Therefore, exploring the interactive influence of diagnosis and *APOE* genotypes is important for elucidating the influence of genes on the brain at different stages of AD development. We found that the interactive influence of diagnosis and *APOE* genotype on brain gFCD was located in the ECN and anterior DMN (MPFC). There were opposite alterations of ECN and DMN from CN to MCI in ε4 and ε2 carriers, and, unexpectedly, brain function was decreased in ε2 carriers and increased in ε4 carriers in MCI patients compared to CN individuals (Figure 4). As the DLPFC plays an essential role for executive function and cognition control processing, while the MPFC is related to the self-referential mental activity (Andrews-Hanna et al., 2014), we speculate that ε2 and ε4 alleles play opposite roles in executive control and self-referential mental processing during the AD development.

Importantly, the mediation analyses showed that the link between *APOE* genotype and cognitive performances was mediated through the hippocampal volume and gFCD in the thalamus in the MCI group. In other words, the protective outcome in *APOE* ε2 carriers and detrimental influence in ε4 carriers on cognitive performance might be attributed to their different influences on hippocampal volume and thalamus function in MCI. In particular, even the direct association between *APOE* and immediate memory is not significant, and the *APOE* genotype could significantly influence memory performance through its influence on hippocampal volume in the MCI group. The hippocampus is the core region for memory processes, and this region is altered early in the development of cognition decline. Recent evidence indicated that *APOE* might regulate neurogenesis in the hippocampus (Hong et al., 2016; Tensaouti et al., 2018). The thalamus, which integrates multimodal information across different cortical networks, has been considered the hub of the functional network and plays a critical role in cognitive flexibility (Halassa and Kastner, 2017; Hwang et al., 2017). The mediator role of the hippocampus and thalamus in *APOE*-related cognition performances in MCI might indicate the neuroplasticity and cognitive flexibility protection mechanism underlying the *APOE* ε2 allele-mediated outcome on cognition. In this regard, our findings suggest that hippocampal volume and thalamus function could be predictors of cognition performance and potential treatment targets in MCI with different *APOE* genotypes. Thus, we propose that the gene–brain–cognition model could be a useful tool to reveal the neural mechanisms underlying the gene influence on cognition.

The present study has several limitations. First, our study had a small sample size and was a cross-sectional study because the longitudinal data of fMRI are limited in ADNI dataset. We did not include the AD group in the study because of the small number of patients in the AD subgroup with the *APOE* ε2 allele. Second, Iacono and Feltis recently reviewed the newly analyzed data and proposed that the *APOE* ε2 and ε4 alleles have antagonistic pleotropic influences on cognitive performance and brain structure and function across the human lifespan (Iacono and Feltis, 2019). The present study explored only the influence of the *APOE* genotype in elderly individuals; further studies should investigate the influence of *APOE* during different periods of life. Third, the cognition domains in the present study included only episode memory and executive function but did not include declarative memory, attention, and visuospatial function. Therefore, further studies should involve neuropsychological tests with special referential cognition domains. Fourth, homozygous and heterozygous ε2 and ε4 allele participants were pooled together in the present study since there were so few participants in the homozygote groups. We have provided this information in the Supplementary Material. However, we believe that the current study provides a useful starting point for further exploring *APOE*-associated behavior and for imaging investigations in patients with AD.

## Conclusion

In conclusion, the present findings indicate that the *APOE* ε2 allele has specific protective influences on immediate memory, executive function, and hippocampal volume during AD progression. In addition, the hippocampus and thalamus mediate the influence of *APOE* on cognitive performance in MCI. The gene–brain–cognition model may provide novel insights on the complex neural underpinnings of the genetically guided pathogenic mechanisms and progression of AD.

## Data Availability Statement

The datasets generated for this study can be found in the http://www.adni-info.org.

## Ethics Statement

The studies involving human participants were reviewed and approved by the Ethical approval was obtained by the ADNI investigators. The patients/participants provided their written informed consent to participate in this study.

## Author Contributions

LG and CZ designed the study, analyzed, and drafted the manuscript. RX, LL, DL, and BZ revised the manuscript and interpreted the data. All authors read and approved the final manuscript.

## Conflict of Interest

The authors declare that the research was conducted in the absence of any commercial or financial relationships that could be construed as a potential conflict of interest.

## Footnotes

1 adni.loni.usc.edu; accessed on April 10, 2019

2 http://www.adni-info.org/pdfs/adni_protocol_9_19_08.pdf

3 http://adni.loni.usc.edu/data-dictionary-search/?q=avlt

4 http://adni.loni.usc.edu/wp-content/themes/freshnews-dev-v2/documents/clinical/ADNI-1_Protocol.pdf

5 http://surfer.nmr.mgh.harvard.edu/

6 http://www.fil.ion.ucl.ac.uk/spm

7 http://www.brainnetome.org

8 https://afni.nimh.nih.gov/pub/dist/doc/program_help/3dClustSim.html
