# Supplementary material for: The Specific Impact of Apolipoprotein E Epsilon 2 on Cognition and Brain Function in Cognitively Normal Elders and Mild Cognitive Impairment Patients
Source: Front Aging Neurosci. 2020 Jan 28;11:374. doi: 10.3389/fnagi.2019.00374 (PMC7081769; doi:10.3389/fnagi.2019.00374)
Supplement: Supplementary file 1 [file Data_Sheet_1.pdf]

Supplementary Material

Methods

First, as the interactive influence (Diagnosis × *APOE*) was found in the hippocampus (see the results, Table 1), an additional analysis was conducted to separately detect the potential main and interactive influences of the diagnosis and APOE genotype on the left and right hippocampal volume. Furthermore, the same mediation analyses were performed to investigate the potential mediating influence of the left and right hippocampal volume in the relationship between APOE and cognition in the MCI group.

Second, according to the opposing outcomes of ε2 and ε4 alleles (Shaw et al., 2007), the ε2/ε4 heterozygous genotype influence on cognition and brain is rarely discussed in the previous study. In our sample of participants, 5 participants with the ε2/ε4 genotype were not included in the first analysis, although it would have been interesting to include this group for a preliminary investigation. However, these 5 participants are all in the CN group; therefore, we could not assess the interaction between diagnosis and genotype using the same approach. Here, we used a one-way analysis of variance (ANOVA) to detect group differences in demographic behavioral , brain volume, and gFCD values (in the region of the gene influence and interactive influence of diagnosis and gene) in the cognitively normal group.

Third, as there are only 6 MCI patients homozygous for APOE ε4 and 5 CN patients homozygous for ε4, we pooled together ε4 homozygotes and heterozygotes into a single ε4+ carrier category. However, it would be interesting to conduct a preliminary investigation of the difference among the homozygote and heterozygote groups. Subsequently, a 2 × 4 (2 groups, 4 APOE genotypes) analysis of covariance (ANCOVA) was carried out to compare the demographic data, neuropsychological performances, and brain volume among the groups.

Results

As shown in the **Table S1**, the main influence of diagnosis and *APOE* genotype on each hemisphere of the hippocampus was not significant. A significant interactive influence on the hippocampal volume was found in the right hemisphere (p = 0.039), but not in the left hippocampus volume (p = 0.34). With regard to the mediation analysis, the left and right hippocampal volumes did not have an indirect influence on the relationships between *APOE* genotype and cognitive performance in the MCI group (95% confidence interval include zero).

With regard to the *APOE* ε2/ε4 genotype analysis, as shown in **Table S2,** compared to the CN group with *APOE* ε2/ε4 genotype, the age in the CN group with an *APOE* ε3ε3 genotype was higher (p = 0.03). In addition, the gFCD in the right DLPFC was higher in CN individuals with the *APOE* ε2/ε4 genotype than in CN individuals with the *APOE* ε3/ε4 (and ε4/ε4) genotype (p = 0.02). No other significant group difference was found between the *APOE* ε2/ε4 genotype and other *APOE* genotypes.

With regard to the different *APOE* homozygote and heterozygote genotype analyses, as illustrated in **Table S3**, group differences were found in ADAS13, MMSE, RAVLT-immediate, Trail B test, and hippocampal volume. Further 2 × 4 ANCOVA analyses revealed a significant main influence of diagnosis on cognitive performance, but no main influence of APOE genotype or interactive influence between diagnosis and APOE genotype (**Table S4**).

Table S1. Main and interactive influences of the diagnosis and *APOE* genotype on the left and right hippocampal volume and mean FD.

	CN			MCI			Diagnosis influence	*APOE* influence	Diagnosis × *APOE* influence
	APOE ε2+ (n=19)	*APOE* ε3ε3 (n=39)	*APOE* ε4+ (n=18)	*APOE* ε2+ (n=13)	*APOE* ε3ε3 (n=46)	*APOE* ε4+ (n=42)	*p-value*	*p-value*	*p-value*
Left Hippocampus (ml)	3.45±0.64	3.84±0.59	3.86±0.63	3.59±0.32	3.70±0.61	3.62±0.54	0.56	0.27	0.34
Right Hippocampus (ml)	3.67±1.19	3.55±1.10	3.92±0.98	4.06±0.73	3.69±1.09	3.35±1.34	0.83	0.35	**0.039**
Mean FD of rs-fMRI (mm)	0.53±0.17	0.42±0.18	0.47±0.20	0.45±0.19	0.49±0.25	0.47±0.24	0.83	0.59	0.18

Abbreviations: CN, cognitively normal; MCI, mild cognitive impairment. FD, frame-wise displacement. rs-fMRI, resting-state functional magnetic resonance imaging.

Table S2. Demographic, neuropsychological information, brain volume, and gFCD characteristic in different *APOE* genotype in the cognitively normal group.

	APOE ε2+ (n=19)	*APOE* ε3ε3 (n=39)	*APOE* ε4+ (n=18)	*APOE* ε2ε4 (n=5)
Age	72.05±5.22	75.07±6.39*	71.99±4.50	67.68±4.62
Sex (F/M)	10/9	19/21	8/10	2/3
Years of education	16.89±2.35	15.71±2.59	17.39±1.94	16.20±2.46
ADAS13	11.89±6.56	9.21±3.94	9.25±3.36	8.26±1.87
MMSE	28.63±1.77	28.79±1.39	28.78±1.30	28.00±1.49
RAVLT-immediate	45.47±11.21	43.76±10.05	42.44±8.93	47.20±6.05
RAVLT-learning	5.26±2.02	5.69±2.50	5.88±2.65	5.00±1.58
RAVLT-forgetting (%)	35.31±28.82	39.40±23.33	36.53±23.54	35.33±15.03
Logical Memory	13.79±3.67	13.76±2.84	13.94±2.64	12.00±4.41
Trails B Test (Second)	84.52±45.11	72.84±22.35	90.11±61.41	91.40±33.34
ICV (10^3 ml)	1.49±0.13	1.57±0.18	1.51±0.16	1.54±0.18
Hippocampus (ml)	7.32±1.08	7.44±0.90	7.74±0.72	7.88±0.43
Entorhinal (ml)	3.64±0.96	3.96±0.67	3.81±0.75	3.96±0.64
Fusiform (ml)	17.76±1.96	18.28±2.02	18.07±2.41	18.99±1.94
Left Precentral gyrus	0.24±0.15	0.40±0.18	0.32±0.16	0.34±0.24
Right Precentral gyrus	0.30±0.20	0.51±0.23	0.37±0.14	0.48±0.20

Right Thalamus	0.23±0.24	0.16±0.17	0.17±0.11	0.36±0.32
PCC	0.35±0.27	0.36±0.24	0.43±0.26	0.33±0.18
Right DLPFC	0.74±0.42	0.83±0.38	0.66±0.34*	1.09±0.31
Right MPFC	0.64±0.48	0.64±0.33	0.46±0.29	0.55±0.15

Note: * $p < 0.05$, compared to APOE ε2ε4 group. Abbreviations: ADAS13, Alzheimer's disease assessment scale –13 items cognitive subscale. MMSE, Mini-Mental State Examination; RAVLT, Rey Auditory Verbal Learning Test; ICV, Intracranial Volume. PCC, posterior cingulate cortex; DLPFC, dorsolateral prefrontal cortex; MPFC, medial prefrontal cortex.

Table S3. Demographic, neuropsychological information, and brain volume characteristic in the CN and MCI groups with different *APOE* homozygote and heterozygote genotypes.

	CN				MCI				
	APOE ε2ε3 (n=18)	*APOE* ε3ε3 (n=39)	*APOE* ε3ε4 (n=13)	*APOE* ε4ε4 (n=5)	*APOE* ε2ε3 (n=13)	*APOE* ε3ε3 (n=46)	*APOE* ε3ε4 (n=36)	*APOE* ε4ε4 (n=6)	*p-value*
Age	68.84±6.59	75.07±6.39	72.64±6.32	73.08±9.69	76.63±5.59	72.20±7.73	71.93±5.86	75.05±4.15	0.23
Sex(F/M)	9/9	19/21	6/7	2/3	5/8	24/22	15/21	3/3	0.85
Years of education	16.15±3.08	15.71±2.59	16.62±2.50	15.2±1.10	16.58±2.61	15.95±2.55	15.58±2.64	14.50±4.46	0.49
ADAS13	11.31±3.90	9.21±3.94	6.62±2.16	9.27±3.65	17.81±9.45	13.81±6.25	15.03±6.56	14.00±9.12	**<0.001**
MMSE	28.69±1.38	28.79±1.39	29.38±0.77	28.2±2.17	27.67±1.97	28.10±1.53	28.47±1.54	28.67±1.03	**0.02**

RAVLT-immediate	47.46±8.00	43.76±10.05	45.54±10.88	43.8±8.84	31.92±8.69	35.37±9.84	37.92±10.08	40.00±12.52	**0.001**
RAVLT-learning	5.92±2.25	5.69±2.50	5.31±2.36	5.40±2.07	4.08±2.47	4.95±2.91	4.58±2.72	4.50±2.74	0.44
RAVLT-forgetting (%)	47.2±33.14	39.40±23.33	53.59±35.19	35.49±16.87	48.15±35.55	52.71±30.67	51.61±27.95	33.44±24.32	0.27
Logical Memory	8.85±4.18	13.76±2.84	9.23±5.25	12.2±3.56	8.50±5.42	6.91±2.81	10.19±4.24	11.33±6.22	0.77
Trails B Test (Second)	80.38±32.74	72.84±22.35	132.23±79.7	74.2±17.05	112.50±44.66	109.93±60.54	90.06±41.16	122.17±83.47	**0.04**
ICV (10^3 ml)	1.57±0.16	1.57±0.18	1.48±0.15	1.49±0.13	1.58±0.15	1.53±0.15	1.49±0.17	1.49±0.19	0.42

Hippocampus (ml)	7.51±0.89	7.44±0.90	7.67±0.83	7.7±0.52	7.28±0.78	7.43±0.86	6.92±0.97	6.79±1.23	**0.03**
Entorhinal (ml)	3.94±0.75	3.96±0.67	3.86±0.84	3.83±0.59	3.61±0.71	3.71±0.55	3.53±0.83	3.59±0.53	0.68
Fusiform (ml)	18.52±1.67	18.28±2.02	18.44±1.5	18.76±2.19	17.33±2.49	17.45±1.83	17.22±2.1	17.31±2.27	0.35

Note: p value is the significance of one-way analysis of variance (ANOVA). Abbreviations: CN, cognitively normal; MCI, mild cognitive impairment; ADAS13, Alzheimer's disease assessment scale –13 items cognitive subscale. MMSE, Mini-Mental State Examination; RAVLT, Rey Auditory Verbal Learning Test; ICV, Intracranial Volume.

Table S4. Main and interactive influences of the diagnosis and *APOE* genotype on cognition and hippocampal volume.

Abbreviations: CN, cognitively normal; MCI, mild cognitive impairment; ADAS13, Alzheimer's disease assessment scale –13 items cognitive

	CN				MCI				Diagnosis	*APOE*	In in
	APOE ε2ε3 (n=18)	*APOE* ε3ε3 (n=39)	*APOE* ε3ε4 (n=13)	*APOE* ε4ε4 (n=5)	*APOE* ε2ε3 (n=13)	*APOE* ε3ε3 (n=46)	*APOE* ε3ε4 (n=36)	*APOE* ε4ε4 (n=6)	*p-value*	*p-* value	*p-*
ADAS13	11.31±3.90	9.21±3.94	6.62±2.16	9.27±3.65	17.81±9.45	13.81±6.25	15.03±6.56	14.00±9.12	<0.001	0.72	0.
MMSE	28.69±1.38	28.79±1.39	29.38±0.77	28.2±2.17	27.67±1.97	28.10±1.53	28.47±1.54	28.67±1.03	0.01	0.73	0.
RAVLT-immediate	47.46±8.00	43.76±10.05	45.54±10.88	43.8±8.84	31.92±8.69	35.37±9.84	37.92±10.08	40.00±12.52	<0.001	0.94	0.
Trails B Test (Second)	80.38±32.74	72.84±22.35	132.23±79.7	74.2±17.05	112.50±44.66	109.93±60.54	90.06±41.16	122.17±83.47	0.03	0.19	0.
Hippocampal volume (ml)	7.51±0.89	7.44±0.90	7.67±0.83	7.7±0.52	7.28±0.78	7.43±0.86	6.92±0.97	6.79±1.23	0.36	0.68	0.

subscale. MMSE, Mini-Mental State Examination; RAVLT, Rey Auditory Verbal Learning Test; ICV.
